# What Is the Clinical Benefit of Neutrophil-Lymphocyte Ratio in Cardiovascular Patients?

**DOI:** 10.5681/jcvtr.2014.028

**Published:** 2014-06-30

**Authors:** Yavuzer Koza

**Affiliations:** Ataturk University Faculty of Medicine, Department of Cardiology, Erzurum, Turkey

**Dear Editor,**

Neutrophil-lymphocyte ratio (NLR) has gained a significant interest in the recent years, especially in cardiovascular research area. NLR is an easy to use parameter with not requiring an extreme effort to obtain. It can be obtained easily from the complete blood count (CBC) test, which is the most performed test in hospitals.

NLR is used to predict cognitive dysfunction after carotid endarterectomy^1^, peripheral arterial disease prognostication^2^, presence, severity and extent of coronary artery disease^3,4^, and calcific aortic stenosis^5^. NLR has also been used for prediction of coronary heart disease mortality^6^, left atrial trombus^7^, impaired myocardial perfusion^8^, outcomes of cryoballoon-based atrial fibrillation ablation^9^, and prognostication of ST-elevation myocardial infarction (STEMI).^10^ In almost all of these studies, higher NLR was associated with worse clinical outcomes in patients with coronary artery disease, STEMI, peripheral arterial disease, calcific aortic stenosis and atrial fibrillation. However, there is no a universally accepted cut-off value of NLR that dictates a negative outcome as normal or abnormal. All of the above-mentioned studies have used different cutt-off values as normal or acceptable. Arbel et al. state >3 as an abnormally high value, other authors vary between <1.4 and <6 being normal.^1-10^

In a recent study, Ghaffari et al.^11^ evaluated the predictive value of peripheral neutrophil count and NLR in determining the prognosis of MI and the risk of major post-MI adverse events. In view of previous studies, it was not surprising to find an association between NLR and the frequency of heart failure and development of ventricular arrhythmias within the first day. The NLR was not associated with mortality but higher neutrophil count was the best predictive value for both mortality and heart failure.

In STEMI, peripheral leukocyte count usually increases within 2 hours after the onset of chest pain, that peaks 2 to 4 days after infarction, returning to normal in one week.^12^ Ghaffari et al.^11^ measured CBC within 12-24 hours of onset of sypmtoms. The shorter lifespan with a rapid turn-over of neutrophils may have also affected the results of the study. Thus, serial neutrophil count may potentially be advantageous over its single measurement at the time of admission. This could easily be examined from the blood samples that are routinely obtained for monitoring cardiac enzymes. Also the measurement of NLR can be potentially affected by conditions such as metabolic syndrome, valvular heart disease, abnormal thyroid function tests, renal or hepatic dysfunction, local or systemic infection and ingestion of anti-inflammatory drugs.^13^

Park et al.^10^ evaluated the prognostic value of leukocyte profiles (neutrophil, lymphocyte, and monocyte absolute counts) in patients with STEMI treated with primary percutaneous intervention (PCI) and found that patients with 24-hour NLRs ≥5.44 were at increased risk for mortality and not admission leukocyte profile but the leukocyte profile at 24 hours after admission was associated with clinical outcomes (all-cause death). Indeed, Chia et al. showed that total leukocyte and neutrophil counts at 24 hours after primary PCI was an independent predictor of adverse cardiac events in patients with STEMI, but not the baseline hematologic indexes.^14^

After bare metal stent implantation hypersensitivity-mediated inflammation by eosinophilic cationic protein may be responsible for major cardiac events.^15^ Furthermore, hypersensitivity reactions against the drug eluting stent polymer material or the eluted drug have been associated with thrombotic complications probably mediated by persistent inflammation and incomplete apposed stent struts.^16^ Ghaffari et al. did not give any information about their revascularisation strategies. Therefore, it is not clear what type of stents were generally used and how long was their door to needle time. Also there was no any information about the patients’ medication.

In conclusion, should the presence of a higher NLR without a universally accepted cutt-off value alert the physician to a higher hospital mortality? If so, how can we manipulate the NLR to improve the outcome? The NLR may be useful when combined with standardized clinical mortality risk prediction scores and other inflammatory markers. More importantly, further epidemiological studies will be required to consider NLR as a useful marker in these clinical settings.

## Ethical issues

Not applicable.

## Competing interests

None.

## References

[1] Halazun HJ, Mergeche JL, Mallon KA, Connolly ES, Heyer EJ (2014). Neutrophil-lymphocyte ratio as a predictor of cognitive dysfunction in carotid endarterectomy patients. J Vasc Surg.

[2] González-Fajardo JA, Brizuela-Sanz JA, Aguirre-Gervás B, Merino-Díaz B, Del Río-Solá L, Martín-Pedrosa M (2014). Prognostic Significance of an Elevated Neutrophil Lymphocyte Ratio in the Amputation-free Survival of Patients with Chronic Critical Limb Ischemia. Ann Vasc Surg.

[3] Açar G, Fidan S, Uslu ZA, Turkday S, Avci AX, Alizade E (2014 Feb 19). Relationship of Neutrophil-Lymphocyte Ratio With the Presence, Severity, and Extent of Coronary Atherosclerosis Detected by Coronary Computed Tomography Angiography. Angiology.

[4] Arbel Y, Finkelstein A, Halkin A, Birati EY, Revivo M, Zuzut M (2012). Neutrophil/lymphocyte ratio is related to the severity of coronary artery disease and clinical outcome in patients undergoing angiography. Atherosclerosis.

[5] Avci A, Elnur A, Göksel A, Serdar F, Servet I, Atilla K (2014 Feb 14). The Relationship between Neutrophil/Lymphocyte Ratio and Calcific Aortic Stenosis. Echocardiography.

[6] Shah N, Parikh V, Patel N, Patel N, Badheka A, Deshmukh A (2014). Neutrophil lymphocyte ratio significantly improves the Framingham risk score in prediction of coronary heart disease mortality: Insights from the National Health and Nutrition Examination Survey-III. Int J Cardiol.

[7] Yalcin M, Aparci M, Uz O, Isilak Z, Balta S, Dogan M (2013 Sep 19). Neutrophil-Lymphocyte Ratio May Predict Left Atrial Thrombus in Patients With Nonvalvular Atrial Fibrillation. Clin Appl Thromb Hemost.

[8] Williams BA, Merhige ME (2013). Association between neutrophil-lymphocyte ratio and impaired myocardial perfusion in patients with known or suspected coronary disease. Heart Lung.

[9] Canpolat U, Aytemir K, Yorgun H, Şahiner L, Kaya EB, Kabakçı G (2013). Role of preablation neutrophil/lymphocyte ratio on outcomes of cryoballoon-based atrial fibrillation ablation. Am J Cardiol.

[10] Park JJ, Jang HJ, Oh IY, Yoon CH, Suh JW, Cho YS (2013). Prognostic value of neutrophil to lymphocyte ratio in patients presenting with ST-elevation myocardial infarction undergoing primary percutaneous coronary intervention. Am J Cardiol.

[11] Ghaffari S, Nadiri M, Pourafkari L, Sepehrvand N, Movasagpoor A, Rahmatvand N (2014). The predictive Value of Total Neutrophil Count and Neutrophil/Lymphocyte Ratio in Predicting In-hospital Mortality and Complications after STEMI. J Cardiovasc Thorac Res.

[12] Sabatine MS, Morrow DA, Cannon CP, Murphy SA, Demopoulos LA, DiBattiste PM (2002). Relationship between baseline white blood cell count and degree of coronary artery disease and mortality in patients with acute coronary syndromes: a TACTICS-TIMI 18 (Treat Angina with Aggrastat and determine Cost of Therapy with an Invasive or Conservative Strategy-Thrombolysis in Myocardial Infarction 18 trial) substudy. J Am Coll Cardiol.

[13] Pearson TA, Mensah GA, Alexander RW, Anderson JL, Cannon RO, Criqui M (2003). Markers of inflammation and cardiovascular disease: application to clinical and public health practice: A statement for healthcare professionals from the Centers for Disease Control and Prevention and the American Heart Association. Circulation.

[14] Chia S, Nagurney JT, Brown DF, Raffel OC, Bamberg F, Senatore F (2009). Association of leukocyte and neutrophil counts with infarct size, left ventricular function and outcomes after percutaneous coronary intervention for ST-elevation myocardial infarction. Am J Cardiol.

[15] Niccoli G, Sgueglia GA, Conte M, Cosentino N, Minelli S, Belloni F (2011). Eosinophil cationic protein and clinical outcome after bare metal stent implantation. Atherosclerosis.

[16] Joner M, Finn AV, Farb A, Mont EK, Kolodgie FD, Ladich E (2006). Pathology of drug-eluting stents in humans: delayed healing and late thrombotic risk. J Am Coll Cardiol.

